# Supplementary material for: Evaluating Leukocyte Telomere Length and Myeloid-Derived Suppressor Cells as Biomarkers for Prostate Cancer
Source: Cancers (Basel). 2024 Mar 31;16(7):1386. doi: 10.3390/cancers16071386 (PMC11011111; doi:10.3390/cancers16071386)
Supplement: Supplementary file 1 [file cancers-16-01386-s001.zip › cancers-2912720-supplementary.pdf]

Supplementary Materials:

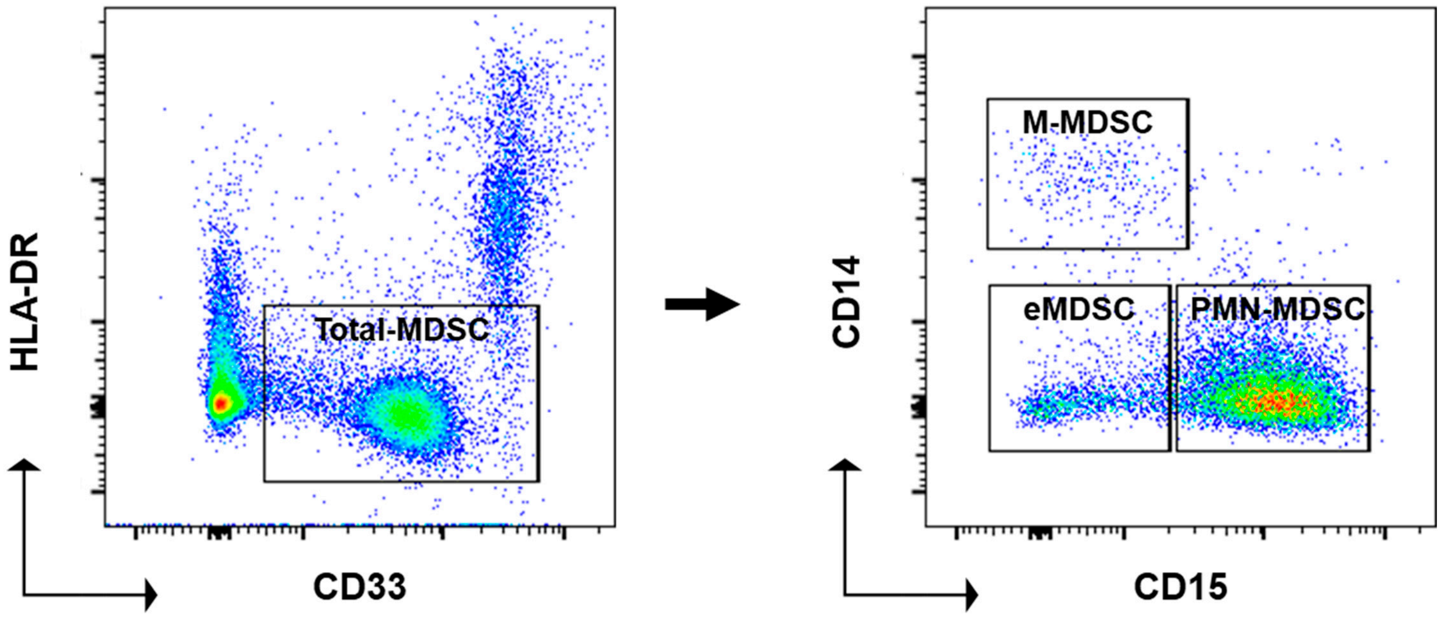

Figure S1. Gating strategy for the identification of MDSC subsets and example of flow cytometry data. Total-MDSC, PMN-MDSC, M-MDSC and eMDSC were characterized as HLA-DR $^{low/-}$ $CD33^+$, HLA-DR $^{low/-}$ $CD33^+$ $CD15^+$ $CD14^-$, HLA-DR $^{low/-}$ $CD33^+$ $CD15^-$ $CD14^+$, and HLA-DR $^{low/-}$ $CD33^+$ $CD15^-$ $CD14^-$ respectively. Each population of MDSC subsets was presented as a percentage of the total PBMC.

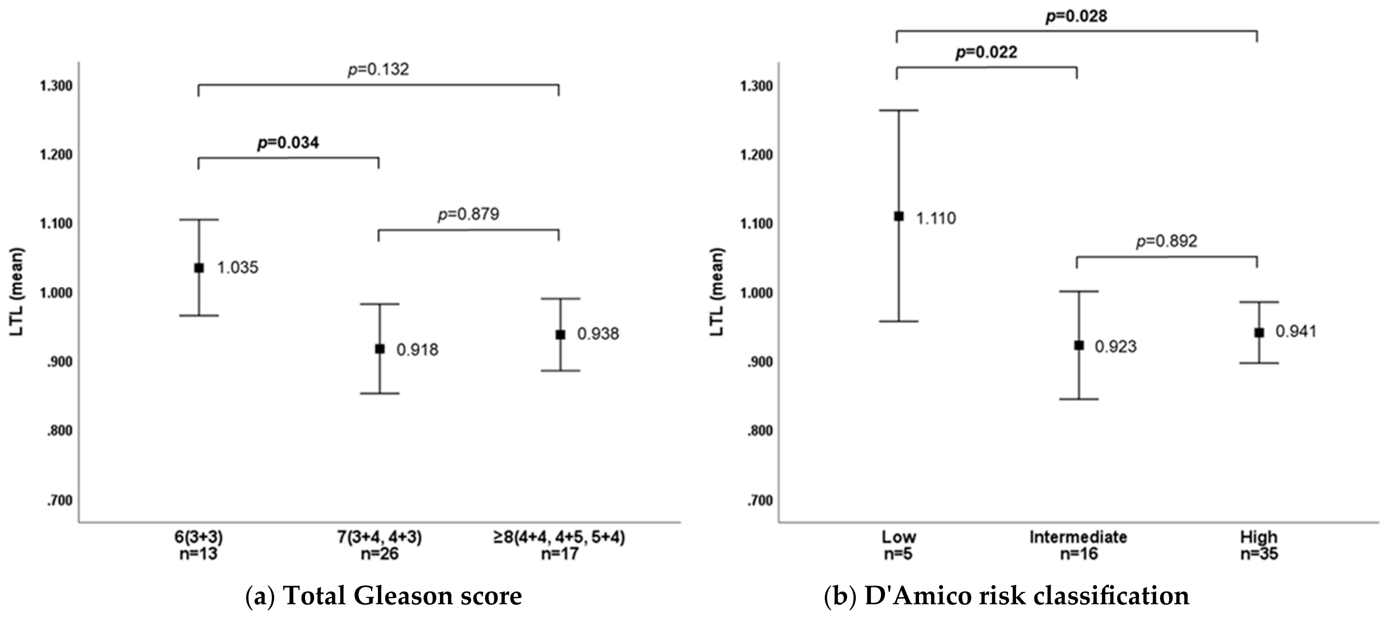

Figure S2. Differences in LTL between groups classified by Total Gleason score (a) or D'Amico risk classification(b).

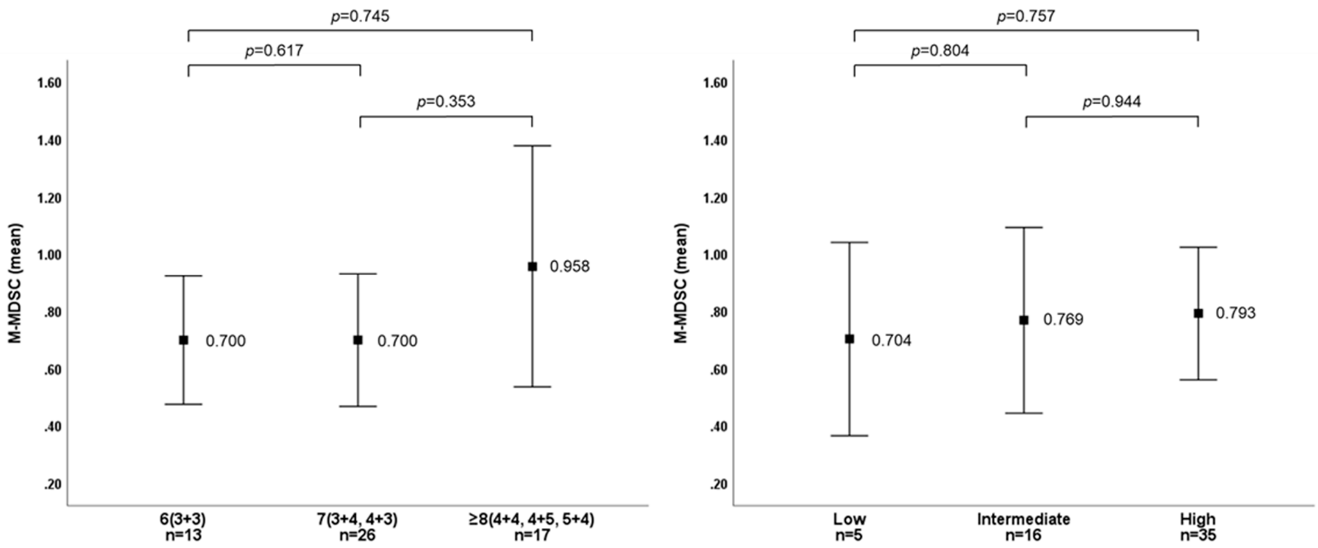

(**a**) **Total Gleason score** (**b**) **D'Amico risk classification**

Figure S3. Differences in M-MDSC between groups classified by Total Gleason score (a) or D'Amico risk classification(b).

Table S1. Baseline characteristics of patients with biopsy results

Characteristics	Total (N=102)		Cancer (N=56)				Control (N=46)				Mean difference (t-test / M-W)		
	Mean / n (%)	SD	Mean / n (%)	Min	Max	SD	Mean / n (%)	Min	Max	SD	t / Z	df	*P*-value
WBC (/µl)	5741.0	1328.8	5791.1	2900	8500	1364.1	5680.4	3400	8600	1296.9	-0.417	100	0.678
Hb (g/dl)	14.3	1.4	14.1	10.3	17.1	1.4	14.6	11.2	17.2	1.4	-2.056	-	**0.040***
Alb (g/dl)	4.2	0.3	4.1	3.4	4.8	0.1	4.2	3.5	4.8	0.1	1.949	100	0.054
Cre (mg/dl)	0.95	0.52	0.96	0.59	5.57	0.42	0.93	0.65	2.43	0.32	0.262	-	0.793*
eGFR (mL/min/1.73 m^2)	68.1	17.0	67.4	8.5	101.3	17.6	68.9	21.5	93.5	16.4	-0.780	-	0.435*
cystatin C (mg/l)	1.04	0.57	1.10	0.57	5.98	0.71	0.96	0.66	2.49	0.32	1.800	-	0.072*
PSA (ng/ml)													
<10 ng/ml, n (%)	73 (71.6)	-	38 (67.9)	-	-	-	35 (76.1)	-	-	-	-	-	-
10–20 ng/ml, n (%)	22 (21.6)	-	12 (21.4)	-	-	-	10 (21.7)	-	-	-	-	-	-
>20 ng/ml, n (%)	7 (6.8)	-	6 (10.7)	-	-	-	1 (2.2)	-	-	-	-	-	-
Total Gleason score													
6(3+3), n (%)	-	-	13 (23.2)	-	-	-	-	-	-	-	-	-	-
7(3+4, 4+3), n (%)	-	-	26 (46.4)	-	-	-	-	-	-	-	-	-	-
≥8(4+4, 4+5, 5+4), n (%)	-	-	17 (30.3)	-	-	-	-	-	-	-	-	-	-
D'Amico risk classification													
Low, n (%)	-	-	5 (8.9)	-	-	-	-	-	-	-	-	-	-
Intermediate, n (%)	-	-	16 (28.6)	-	-	-	-	-	-	-	-	-	-
High, n (%)	-	-	35 (62.5)	-	-	-	-	-	-	-	-	-	-
Hypertension, n (%)	57 (55.9)	-	30 (53.6)	-	-	-	27 (58.7)	-	-	-	-	-	0.604**
Diabetes mellitus, n (%)	14 (13.7)	-	6 (10.7)	-	-	-	8 (17.4)	-	-	-	-	-	0.330**
Dyslipidemia, n (%)	35 (34.3)	-	18 (32.1)	-	-	-	17 (37.0)	-	-	-	-	-	0.610**
Hyperuricemia, n (%)	22 (21.6)	-	17 (30.4)	-	-	-	5 (10.9)	-	-	-	-	-	**0.017****
Coronary artery disease, n (%)	4 (3.8)	-	3 (5.4)	-	-	-	1 (2.2)	-	-	-	-	-	0.410**
Cerebrovascular disease, n (%)	4 (3.8)	-	2 (3.6)	-	-	-	2 (4.3)	-	-	-	-	-	0.841**

* Due to their non-normal distributions, the assessment was conducted using the Mann-Whitney U-test.

** The assessment was conducted the Chi-square test.

Table S2. Correlation with LTL

Variables	r / ρ	*P*-value
Total-MDSC (%/PBMC)	-0.047	0.641*
PMN-MDSC (%/PBMC)	-0.167	0.093*
Log PMN-MDSC	-0.165	0.097
M-MDSC (%/PBMC)	-0.346	**<0.001***
Log M-MDSC	-0.373	**<0.001**
Age (years)	-0.179	0.071
BMI (kg/m2)	-0.127	0.205
WBC (/μl)	-0.080	0.426
Hb (g/dl)	-0.056	0.578*
Alb (g/dl)	0.045	0.655
Cre (mg/dl)	0.029	0.772*
eGFR (mL/min/1.73m^2)	0.011	0.914*
cystatin C (mg/l)	-0.202	**0.042***
HbA1c (%)	-0.292	**0.006***
Teststerone (ng/mL)	0.140	0.161
PSA (ng/ml)	-0.079	0.432*
CRP (mg/dl)	-0.114	0.252*

* Due to their non-normal distributions, the assessment was conducted using the Spearman's rank correlation coefficient.

Table S3. Correlation with Log M-MDSC

Variables	r / ρ	*P*-value
Total-MDSC (%/PBMC)	0.046	0.649*
PMN-MDSC (%/PBMC)	0.157	0.116*
Log PMN-MDSC	0.144	0.149
Age (years)	0.056	0.576
BMI (kg/m2)	0.152	0.127
WBC (/μl)	0.239	**0.016**
Hb (g/dl)	0.048	0.631*
Alb (g/dl)	0.089	0.372
Cre (mg/dl)	-0.051	0.613*
eGFR (mL/min/1.73m^2)	0.047	0.638*
cystatin C (mg/l)	0.110	0.269*
HbA1c (%)	0.174	0.107*
Teststerone (ng/mL)	-0.123	0.219
PSA (ng/ml)	0.030	0.768*
CRP (mg/dl)	0.266	**0.007***

* Due to their non-normal distributions, the assessment was conducted using the Spearman's rank correlation coefficient.
